# *Hot start* reverse transcriptase: an approach for improved real-time RT-PCR performance

**DOI:** 10.1186/s40543-015-0063-4

**Published:** 2015-06-21

**Authors:** Nils Rutschke, Jan Zimmermann, Ronny Möller, Gerd Klöck, Mathias Winterhalter, Annika Leune

**Affiliations:** 1 altona Diagnostics GmbH, Moerkenstr. 12, 22767 Hamburg, Germany; 2 Institute of Environmental Biology and Biotechnology, University of Applied Sciences Bremen, Am Neustadtswall 30, 28199 Bremen, Germany; 3 School of Engineering and Science, Jacobs University, Campus Ring 1, 28759 Bremen, Germany

**Keywords:** Real-time RT-PCR, Reverse transcriptase, Hot start, MERS-CoV

## Abstract

**Background:**

Reverse transcriptase is an indispensable enzyme for real-time reverse transcriptase (RT)-PCR, a standard method in molecular diagnostics for detection and quantification of defined RNA molecules. The prevention of non-specific products due to elongation of misprimed oligonucleotides by the enzyme at temperatures beneath the specific annealing temperature is one of the biggest challenges in real-time RT-PCR.

In the present study, an aptamer directed against the reverse transcriptase was analyzed for its potential to attain a temperature-dependent reverse transcriptase (“hot start” RT).

**Findings:**

The hot start effect was investigated in a one-step real-time RT-PCR assay for the detection of Middle East respiratory syndrome coronavirus (MERS-CoV). Results with aptamer revealed a reduced RT activity at low temperatures while achieving full activity at the specific annealing temperature of 55 °C. Sensitivity (limit of detection (LoD) 95 %) of the MERS-CoV assay was increased by about two times in the presence of aptamer.

**Conclusions:**

The study demonstrates the potential of aptamer-dependent hot start RT for the improvement of diagnostic real-time RT-PCR assays.

## Findings

### Introduction

Real-time RT-PCR is the method of choice in molecular diagnostics for detection and quantification of defined RNA molecules (Mackay 2004). This technique utilizes reverse transcriptase (RT) to convert RNA into complementary DNA (cDNA), a thermostable DNA-dependent DNA polymerase for the amplification of specific target sequences and target specific probes (oligonucleotides) labelled with fluorophores for the detection of amplified DNA (Gibson et al. 1996).

Real-time RT-PCR is regarded as a method with high sensitivity and specificity (Martel et al. 2002). However, this is challenged by non-specific products generated by elongation of misprimed primer that competes with the synthesis of specific amplification products in each cycle (Chou et al. 1992; Li et al. 1990). The probability of non-specific product formation increases with the complexity of the real-time RT-PCR system and the background nucleic acid in the specimen (Brownie et al. 1997, Handschur et al. 2009). Ultimately, non-specific products can severely decrease sensitivity as well as specificity of real-time RT-PCR assays (Sharkey et al. 1994; Birch et al. 1996; Jayasena 1999).

Assays for the detection of RNA viruses are often highly complex (high quantity of different oligonucleotides) due to low sequence conservation of the RNA genome (Gardner et al. 2004; Brownie et al. 1997). In general, mispriming occurs at temperatures below the specific annealing temperature of the oligonucleotides (Jayasena 1999). Thus, the formation of non-specific products can be reduced by using hot start variants of the enzymes, which are inactive at low temperatures and activated at higher temperatures, appropriate for specific primer annealing to the target nucleic acid (Birch et al. 1996).

Several biological or chemical hot start concepts exist for *Taq* polymerase, a thermostable DNA-dependent DNA polymerases. The *Taq* polymerase can be inactivated by binding of specific antibodies or aptamers, by incubation with chemicals or by altered molecular kinetics (Sharkey et al. 1994; Birch et al. 1996, Hermann and Patel 2000; Gening et al*.* 2006; Kermekchiev et al. 2003). The activation is obtained by heating up to ≥95 °C during the initial denaturation step of the PCR. In contrast to *Taq* polymerase, which is heat-stable up to temperatures of ≥95 °C, the RT is only stable at temperatures ranging from 42 to 70 °C (Pfaffl 2010; Gallup 2011). Therefore, other hot start concepts for the reversible inactivation of reverse transcriptase need to be developed.

A high-affinity RNA ligand (aptamer), which targets moloney murine leukemia virus (M-MLV) RT was described by Chen and Gold (1994). The aptamer is assumed to inactivate the RT by blocking the nucleic acid binding site of the RT (Chen and Gold 1994; Chen et al. 1996).

In the present study, the aptamer was analyzed in a one-step real-time RT-PCR assay for the detection of *Middle East respiratory syndrome coronavirus* (MERS-CoV) to investigate the potential of a hot start RT for improved real-time RT-PCR performance. MERS-CoV was first identified in 2012 (Zaki et al. 2012). Since the discovery, the World Health Organization noted 971 cases of MERS-CoV, and of these, 365 led to a lethal outcome (WHO 2015). Therefore, a more sensitive detection system, especially in the presence of low viral loads in patients, would be favorable for an early diagnosis and treatment.

### Material and methods

#### Real-time RT-PCR setup

The one-step real-time RT-PCR was performed in a 25-μL reaction mix containing 10 μL of RNA template, 1x PCR reaction buffer (altona Diagnostics GmbH), 2.4 mM MgCl_2_ (Sigma-Aldrich), 240 μg/μL BSA (Roche), 1 U of Platinum® *Taq* DNA Polymerase high fidelity (Invitrogen), 156 U of SuperScript® III Reverse Transcriptase (Invitrogen).

The MERS-CoV specific primer and probe, targeting the genomic region upstream of the *Envelope* gene (upE), were synthesized as published (Corman et al. 2012a; Corman et al. 2012b). The RT-PCR reaction included 0.8 μM of primer UpE-Fwd (GCAACGCGCGATTCAGTT), 0.8 μM of primer UpE-Rev (GCCTCTACACGGGACCCATA), and 0.1 μM of probe (FAM-CTCTTCACATAATCGCCCCGAGCTCG-TAMRA).

Thermal cycling conditions were 55 °C for 20 min (RT-step), 2 min at 95 °C (*Taq* polymerase activation and RT inactivation), followed by 45 cycles of 15 s at 95 °C (denaturation), 45 s at 58 °C (annealing and acquisition) and 15 s at 72 °C (extension). In case of thermal gradient real-time RT-PCR, the RT-step was performed in parallel at 31 °C for one part and 55 °C for the other part of the 96-well plate.

All real-time RT-PCRs were performed on a CFX96 Touch™ Real-Time PCR Detection System (BioRad).

#### Aptamer

The aptamer (5′-CUUACCACGCGCUCUUAACUGCUAGCGCCAUGGCCAAAACU-3′) published by Chen and Gold (1994) was synthesized at altona Diagnostics GmbH. A 3′ phosphorylation was added to the aptamer to eliminate any possible elongation of the aptamer.

The reverse transcriptase was incubated with increasing concentrations (0, 12.5, 25, 50, 100, or 200 μM) of aptamer for 15 min at room temperature (20 °C) before adding to the reaction mix.

To demonstrate an aptamer-dependent hot start RT effect, the RT-step was carried out in parallel at 55 °C, the specific annealing temperature of the primer and probe, and at 31 °C, at which the RT shows a significant activity but which is below the specific annealing temperature of the primer and probe. Each aptamer concentration was analyzed in three replicates.

#### Real-time RT-PCR template

An *in vitro* transcribed RNA (IVT) based on a sequence of MERS-CoV strain EMC/2012 was used as real-time RT-PCR template. The concentration of the IVT was determined by spectrophotometry.

#### Valuation of analytic sensitivity

The analytic sensitivity (limit of detection (LoD)) is defined as the concentration (copies/reaction) of MERS-CoV specific RNA (IVT) molecules that can be detected with a positive rate of ≥95 %. The analytic sensitivity was determined by analyzing a half-logarithmic serial dilution of the IVT ranging from 100 to 0.1 copies/reaction. Each concentration was analyzed four times in six replicates (*n* = 24). Hit rates were subjected to probit regression and correlation analysis in StatsDirect software (Version 2,7,9; StatsDirect statistical software).

The RT was either incubated with 25 μM aptamer or without aptamer for 15 min at room temperature (20 °C), before adding to the reaction mix.

### Results

#### Aptamer leads to hot start effect

The reverse transcriptase was incubated with different concentrations of the aptamer (0, 12.5, 25, 50, 100, and 200 μM per reaction) before adding to the reaction mix. The RT-step was performed in parallel at 55 and 31 °C.

**Fig. 1:**
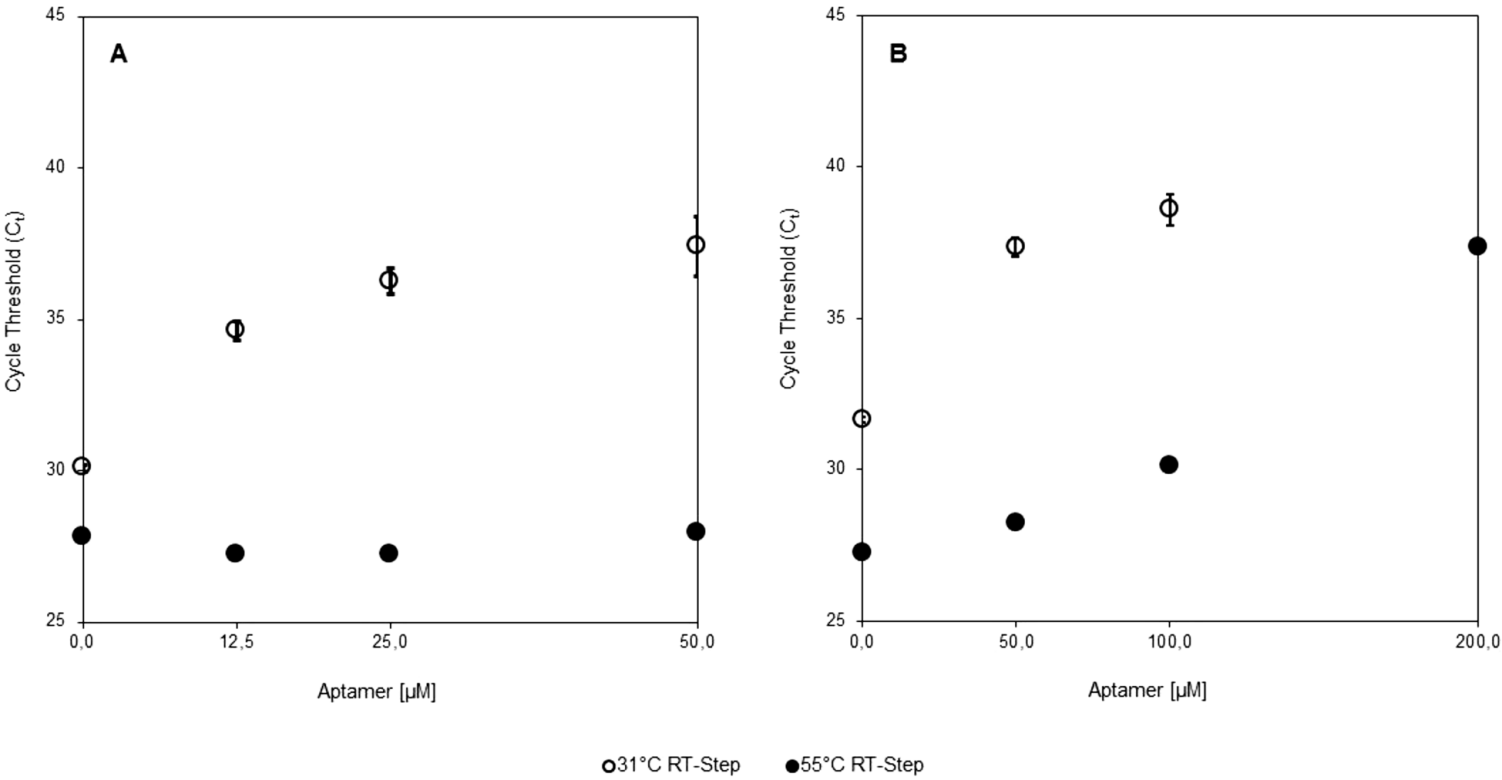
Relation between aptamer concentration and cycle threshold (*C*_*t*_) at 31 and 55 °C. Aptamer concentrations of 0, 12.5, 25, and 50 μM per reaction (**a**) and 0, 50, 100, and 200 μM per reaction (**b**) were tested in a one-step real-time RT-PCR assay for the detection of Middle East respiratory syndrome coronavirus (MERS-CoV) using 1000 RNA copies per reaction. RT-step was carried out for 20 min in parallel at 31 °C (*circles*) and 55 °C (*dots*), respectively. Results of 200 μM aptamer concentration at 31 °C did not lead to a positive signal and therefore are not shown

Incubation of RT with aptamer and RT-step at 31 °C resulted in a mean delayed cycle threshold of up to ∆*C*_*t*_ 7.31 (50 μM aptamer, Fig. 1a) compared to the reference without aptamer, indicating reduced RT activity (Fig. 1a, b).

The results can be summarized as, the higher the aptamer concentration the later the amplification signal. Two hundred micromolar of aptamer revealed no amplification signal at all.

The RT-step at 55 °C, with 12.5 and 25 μM aptamer showed slightly earlier cycle thresholds compared to the reference without aptamer (∆*C*_*t*_ 0.60 and 0.61), while concentrations of 50 μM and higher resulted in delayed amplification signals, compared to the reference without aptamer (Fig. 1a, b).

Since the aptamer concentration of 25 μM allowed full RT activity at 55 °C but significantly reduced RT activity at 31 °C, this concentration was chosen to survey the influence of the aptamer on the analytic sensitivity of the MERS-CoV assay.

#### Evaluation of hot start RT for improved detection of a low copy number target

In order to investigate, if the hot start RT lead to an increase in analytical sensitivity of the MERS-CoV assay, a half-logarithmic serial dilution of the MERS-CoV IVT ranging from 100 to 0.1 copies per reaction was analyzed with a reaction mix containing hot start RT (25 μM aptamer) and standard RT (without aptamer).

**Fig. 2:**
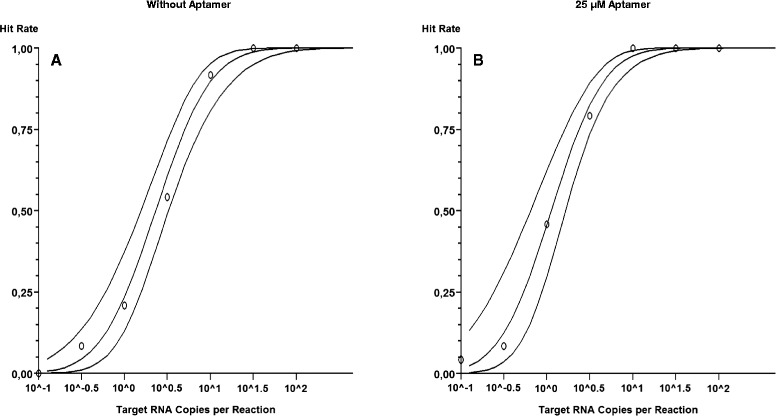
Probit regression analyses of a real-time RT-PCR with 25 μM aptamer and without aptamer. Probit regression analysis was carried out with target RNA loads from 10^−1^ to 10^2^ copies per reaction. Each dilution was analyzed in four independent runs with six replicates. The target RNA concentration is plotted on the X-axis, and the Y-axis displays the hit rate. *Circles* are experimental data points; the *inner lines* represent the corresponding probit curve, *outer lines* the 95 % confidence intervals. **a** Without aptamer; **b** 25 μM aptamer

Real-time RT-PCR was carried out to determine concentration-dependent hit rates (Table 1), which were analyzed in a probit regression (Fig. 2).

**Table 1:** Hit rate of 25 μM aptamer and without aptamer in real-time RT-PCR MERS-CoV assay. Half-logarithmic serial dilutions of MERS-CoV RNA, ranging from 10^−1^ to 10^2^ copies per reaction were analyzed. The ratio of positive signals to all signals, followed by the data in percentage is given below

	Copies (MERS-CoV IVT)/reaction						
	10^2^	10^1.5^	10^1^	10^0.5^	10^0^	10^−0.5^	10^−1^
Aptamer (25 μM/reaction)	24/24	24/24	24/24	19/24	11/24	2/24	1/24
	100 %	100 %	100 %	79.17 %	45.83 %	8.33 %	4.17 %
Control (without aptamer)	24/24	24/24	22/24	13/24	5/24	2/24	0/24
	100 %	100 %	91.67 %	54.17 %	20.83 %	8.33 %	0 %

The MERS-CoV assay with the hot start RT has a LoD of 6.9 copies/reaction (95 % confidence interval (CI): 4.2–15.4 copies/reaction), whereas the assay without aptamer has a LoD of 15.5 copies/reaction (95 % confidence interval (CI): 9.3–34.7 copies/reaction) (Fig. 2a, b).

### Conclusion

Hot start *Taq* polymerases have proven to be valuable tools to improve analytical sensitivity and specificity in real-time PCR assays, by reducing non-specific products.

Based on this experience, the idea arose to improve the performance of real-time RT-PCR assays by developing a hot start concept for the reverse transcriptase.

In this study, we demonstrated that a hot start RT can be generated by using an aptamer directed against M-MLV RT.

The use of this hot start RT in a MERS-CoV assay led to an increase of the analytical sensitivity of about twofold compared to the analytical sensitivity of the same assay with standard RT.

In summary, we could demonstrate that hot start RT has the potential to improve the sensitivity of real-time RT-PCR assays.

This finding will be of special interest for more complex assays or for assays which are used with specimen with a high load of non-target nucleic acids in routine molecular diagnostics.
